# Financial Awards and Their Effect on Football Players’ Anxiety and Coping Skills

**DOI:** 10.3389/fpsyg.2020.01148

**Published:** 2020-06-10

**Authors:** Adriana Kaplánová

**Affiliations:** Faculty of Physical Education and Sports, Comenius University, Bratislava, Slovakia

**Keywords:** emotions, mental preparation, stress management, salary, mediation analysis, sports performance, sports psychology

## Abstract

**Objective:**

Financial awards can be an important factor affecting athletes’ mental preparation and various skills to manage stress. Since such a link has not yet been studied, the study has been designed to evaluate the moderation effect of financial awards in relation to football players’ anxiety and coping skills.

**Methods:**

The study consists of 110 male football players aged 18–32 years old (mean ± SD: 23.98 ± 3.01 years) who were divided into two groups: financial awarded (*n* = 48) and financial unawarded for sports performance (*n* = 62). The anxiety of football players was measured by the Sport Anxiety Scale SAS-2. Coping strategies to manage stress were assessed by the Athletic Coping Skills Inventory ACSI-28. The effect of financial awards in relation to football players’ anxiety and coping skills was evaluated by the mediators’ model using the PROCESS software (Hayes, 2018).

**Results:**

The results suggest that financial awards are important factors that influence football players’ anxiety and coping skills. The financial awards increase the motivation of football players to better prepare for sports performance, which has been proven, through better setting of performance goals and more careful mental preparation. Financially awarded football players seem to respect the coach and follow his instructions to a greater extent than unawarded football players, which may be due to the financial benefits and the commitment they have confirmed by signing to the football club. In another aspect, the financial awards are likely to increase the cognitive trait of the anxiety of football players. It seems that financial players are more concerned about the failure of the match, which increases their anxiety, especially since it is a cognitive part and affects their sports performance.

**Conclusion:**

For this reason, we encourage sports organizations to focus more on the mental preparation of football players. It is important to provide football players the opportunity to graduate from short- or long-term mental training conducted by a trained sports psychologist not only at the time of the athlete’s failure but also as a preventive measure against increasing cognitive anxiety. We recommend sports organizations to train coaches in the field of mental training, preferably through annual short training sessions with a sports psychologist, to influence the development of desirable athletes’ coping skills.

## Introduction

Sports performance is associated with a multitude of various feelings. Sometimes athletes feel the excitement and believe in achieving valued goals that bring them coveted happiness and satisfaction. In another aspect, they can feel also scared due to which they become nervous, their muscles get tense, their stomach pains, the body becomes tight, the hands become clammy, and negative thoughts predominate them and hence they start believing that they will never win (Hanin, 2000; Skinner and Brewer, 2004; Bhambri et al., 2005; Bali, 2015; Kaplánová, 2019a, b).

During the competition, athletes’ emotions are very intense and in some cases may grow into anxiety (Cisler et al., 2010; Ford et al., 2017; Kaplánová, 2019c; Rice et al., 2019). Anxiety is an unpleasant mental state accompanied by a premonition of threat. It usually binds to an object that is non-specific, unnecessary, scattered, or vague (Martens et al., 1990; Rice et al., 2019). According to psychoanalysts, the stressor may be conscious or unconscious conflicts (Connolly, 2018). Some athletes tend to attach deeper importance to conflicts and therefore experience a stressful situation more often than others (Kaplánová, 2019a, b,c). Moreover, not every stressful situation that an athlete experiences is real and the stressor is present. This is due to a phenomenon that is related to the complexity of the human psyche and is also closely related to self-concept (Sapolský, 2012; Kaplánová and Gregor, 2018). Stressors according to the type of threat can be divided into real and potential stressors. While real stressors really threaten and impair the quality of life of a person, potential stressors relate to the ability to visualize the stressors in such quality and intensity that they produce the same physiological response as real stressors (Godoy et al., 2018).

In a sports context, anxiety is defined as an athlete’s tendency to perceive competition as a threat to the organism (Grupe and Nitschke, 2014). While cognitive trait anxiety is associated with negative thinking and fear of sports performance, emotional trait anxiety is associated with the perception of one’s own physiological signs of activation, such as rapid heartbeat, shortness of breath, shaking hands or feet, sweating, muscle stiffness, and others (Martens et al., 1990; Kaplánová, 2019a, c; Rice et al., 2019; Gallegos et al., 2020). Athletes’ emotions are therefore divided into precompetitive, competitive, and postcompetitive (Cerin and Barnett, 2011; Pellizzari et al., 2011). They have the character of circular feedback, of varying intensity, taking place over time and interacting with each other (Paul and Garg, 2012; Gregor, 2013). They are influenced by factors such as the athletes’ mental resilience, their skills to cope with stress, previous experience of a similar situation, expected or unexpected running sports performance, as well as high or low support of fans (Karamousalidis et al., 2006; Kimbrough et al., 2007; Daroglou, 2011; Jooste et al., 2014).

The competition is a culmination of the training of athletes during which they make every effort to achieve the best possible physical and mental effort. Coping skills are the ability of a person to meet conditions that currently exceed their adaptive skills (Hanton et al., 2015). It is an effort of behavioral and cognitive character that is aimed at adapting or overcoming requirements (Folkman and Moskowitz, 2004). Coping skills are generally associated with adaptive, constructive ways of dealing with a stressful situation in which the individual successfully eliminates stress and is able to perform optimally (Géczi et al., 2008; Vidic et al., 2017). The competition is very demanding for the psychological processing of an unexpected result. If athletes are unable to handle it properly, it can lead to various psychological problems. It has been found that there are fragments that consist of fear, tension, or unpleasant experiences that can cumulate and grow into anxiety. Therefore, it makes sense to deal with psychological training of athletes not only when the athletes fail but also as a preventive measure against failure (Kaplánová, 2018; Kaplánová, 2019a, b,c).

Over the past decade, experts have increased research attention to skills to manage stress in various sports (Bebetsos and Antoniou, 2003; Karamousalidis et al., 2006; Daroglou, 2011; Jooste et al., 2013, 2014; Bebetsos, 2015; Kaplánová, 2018, 2019a,b,c; Hernandez et al., 2019). Also, they have increased attention in the field of anxiety research in the context of sports performance (Géczi et al., 2008; Ford et al., 2017; Castro-Sánchez et al., 2019; Kaplánová, 2019a, c; Gallegos et al., 2020). Financial awards can be an important factor affecting athletes’ mental preparation and various skills to manage stress. Since such a link has not yet been studied, the study has been designed to evaluate the moderation effect of financial awards in relation to football players’ anxiety and coping skills. We have defined a research question “Are the financial awards a moderator of the relationship between football players’ anxiety and coping skills?”

## Materials and Methods

### Participants

The study involved 110 male football players aged 18–32 years old (mean ± SD: 23.98 ± 3.01 years) and of sporting age (mean ± SD: 15.25 ± 2.25). At the time of filling in the inventories, all participants were active football players registered in the Slovak Football Association (SFZ). Participants were divided into two groups. The criterion of distribution was the signing of a professional contract with the relevant football club and financial awards for sports performance. The study involved 48 awarded football players aged 18–32 years old (mean ± SD: 23.08 ± 2.74 years) playing in the first football league currently known as the Fortuna Ligue, and 62 unawarded football players aged 19–29 years old (mean ± SD: 23.08 ± 3.36 years) playing in lower football leagues.

### Measures

#### Athletic Coping Skills Inventory ACSI-28

This inventory is a validated tool commonly used in discovering the level of coping skills among athletes (Smith et al., 1995). ACSI-28 is composed of 28 items and 7 subscales. Each statement in ACSI-28 describes the experiences of other athletes, which prompts the participant to indicate the frequency of similar experiences. Whether athletes are able to remain calm, even when things are going badly, is monitored by the subscale of coping with adversity. The subscale of coachability evaluates openness and the ability to listen to the coach’s instructions. Good sports performance in competition is also dependent on concentration, confidence, and achievement motivation, which evaluates the subscale of concentration and the subscale of confidence and achievement motivation. Athletes should be mentally prepared for performance and have their stress under control during the competition. The level of these skills monitors the subscale of goal setting and mental preparation and the subscale of peaking under pressure. Sometimes it happens that athletes worry about what others will think if they perform poorly. These worries assess the subscale called freedom from worry (Smith et al., 1995). The response format for each item consists of a linear four-point scale ranging from 0 (almost never) to 3 (almost always). Scores range from a low of 0 to a high of 12 on each subscale, with higher scores indicating greater strengths on that subscale. The score for the total scale ranges from a low of 0 to a high of 84, with higher scores signifying greater strength. Cronbach’s alpha based on the classical items analysis was from 0.70 to 0.78.

#### Sport Anxiety Scale SAS-2

This scale is a measure that assesses the competitive trait anxiety experienced by athletes. It is a multidimensional measure of anxiety in sports performance settings. It is composed of a 15-item scale and includes three subscales: worry, somatics, and concentration disruption. The cognitive trait anxiety involved concerns about performing poorly and the resulting negative consequences. The somatic construct involved various indices of autonomic arousal centered in the stomach and muscles. And the concentration disruption involved difficulties in focusing on task-relevant cues (Smith et al., 2006). The response format for each item consists of a linear four-point scale ranging from 1 (no at all) to 4 (very much). The score for each subscale is calculated as the mean of the scores of subscale items and varies from one to four, with a low score indicating a less intense form of that type of competitive anxiety and a high score indicating a high probability of exhibiting that type of anxiety. Cronbach’s alpha based on the classical items analysis was 0.78 for cognitive trait anxiety, 0.91 for somatic trait anxiety, and 0.73 for concentration disruption.

### Procedure

Athletics coping skills inventories ACSI-28 and Sport Anxiety Scale SAS-2 were applied by a sports psychologist (author of the study) with years of experience in the field. The inventories were provided in a single booklet, which was given to football players. In a complementary way, data about sociodemographic aspects, information about contracts with football clubs, and financial awards for sports performance were also collected. In all the cases, the questionnaires were filled anonymously, and participation in the study was entirely voluntary. The study design was approved by the Ethics Committee of Comenius University in Bratislava, Slovakia. All participants were informed about the aims, methods of data collection, and their use for research purposes. In addition, all participants gave written informed consent in accordance with the Declaration of Helsinki.

### Statistical Analyses

We subjected our data to regression moderation analysis focusing on the moderation role of financial awards in the relationship between coping skills and anxiety. Computational macro for path analysis is based on moderation providing coefficient estimations using ordinary least squares regression for continuous variables. The moderation effect was estimated using Process 3.1 developed by Hayes (2018). To reduce the chances of obtaining false-positive results (type I errors), Bonferroni correction was additionally conducted (Bland and Altman, 1995). The data were analyzed by using the PROCESS software (Hayes, 2018) implemented on SPSS IBM SPSS Statistics.

## Results

**FIGURE 1:**
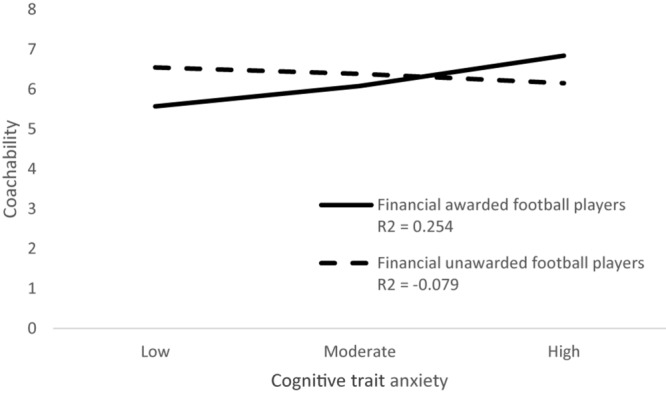
Moderation effect of financial awards in the relationship coachability and cognitive trait anxiety.

**FIGURE 2:**
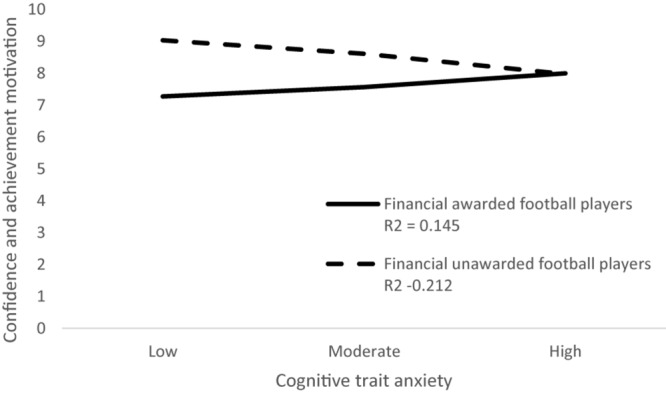
Moderation effect of financial awards in the relationship confidence and achievement motivation and cognitive trait anxiety.

**FIGURE 3:**
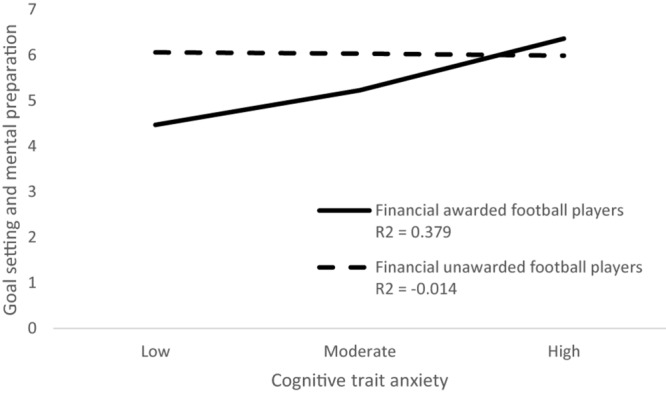
Moderation effect of financial awards in the relationship goal setting and mental reparation and cognitive trait anxiety.

List of study variables with their possible score ranges, mean scores, and standard deviations separately for a group of awarded and unawarded football players are presented in Table 1. The results of moderation analysis with selected coping skills as dependent variables, cognitive trait anxiety as an independent variable, and financial awards as a moderator are presented in Table 2. Together, 21 analyses were carried out. The moderation effect of the financial awards was found to be significant in the subscale coachability of football players and their cognitive trait anxiety [*F*(3,106) = 3.24, *p* < 0.01, *R*^2^ = 0.084]. The moderation effect of financial awards was detected as significant between subscale confidence and achievement motivation and cognitive trait anxiety [*F*(3,106) = 2.91, *p* < 0.05, *R*^2^ = 0.076], as well as between subscale goal setting and mental preparation of football players and their cognitive trait anxiety [*F*(3,106) = 4.64, *p* < 0.05, *R*^2^ = 0.116]. The moderation effect of financial awards in the relationship between selected coping skills of football players and their cognitive trait anxiety is graphically presented in Figures 1–3.

**TABLE 1:** List of study variables with their possible score ranges, mean scores, and standard deviations separately for a group of awarded and unawarded football players.

		**Football players (*n* = 110)**			
		**Awarded (*n* = 48)**		**Unawarded**	
				**(*n* = 62)**	
			
**Variables**	**Ranges**	***M***	**SD**	***M***	**SD**
Coping with adversity	0–12	7.21	1.87	6.55	1.99
Coachability	0–12	6.33	1.62	6.11	1.63
Concentration	0–12	7.42	1.91	7.29	1.84
Confidence and achievement motivation	0–12	8.46	2.02	7.58	2.23
Goal setting and mental preparation	0–12	6.02	2.33	5.27	2.08
Peaking under pressure	0–12	6.48	2.79	5.42	2.78
Freedom from worry	0–12	4.35	2.11	5.26	2.26
Cognitive trait anxiety	5–20	8.90	2.83	10.52	3.62
Emotional trait anxiety	5–20	8.71	2.34	8.13	2.32
Concentration disruption	5.20	5.54	1.71	5.89	1.45

**n*, sample size; Range, possible score range; *M*, mean; SD, standard deviation.*

**TABLE 2:** Results of moderation analysis with selected coping skills as dependent variables, cognitive trait anxiety as the independent variable, and financial awards as the moderator.

**Predictors**	**Coefficient**	**95% Confidence intervals**	***R*^2^ whole model**	***R*^2^ increase due to interaction**
**Model 1 – Coachability as dependent variable**				
CA	0.109	(−0.020, 0.238)	0.084	0.013**
Financial awards	0.182	(−0.422, 0.787)		
CTA × CA	–0.333	(−0.593, −0.073)		
**Model 2 – Confidence and achievement motivation as dependent variables**				
CAM	–0.011	(−0.185, 0.163)	0.076	0.046*
Financial awards	0.910	(0.095, 1.725)		
CTA × CAM	–0.357	(−0.707, −0.006)		
**Model 3 – Goal setting and mental preparation as dependent variables**				
GSMP	0.207	(0.034, 0.380)	0.116	0.028*
Financial awards	0.656	(−0.156, 1.467)		
CTA × GSMP	–0.393	(−0.742, −0.045)		

CA, coachability; CAM, confidence and achievement motivation; GSMP, goal setting and mental preparation; CTA, cognitive trait anxiety. **p ≤ 0.01, *p ≤ 0.05.

## Discussion

We provide evidence that financial awards are important factors affecting football players’ anxiety and coping skills. This result specifies the understanding of the financial award’s effect on their cognitive trait anxiety and selected coping skills. The results of our research extend the knowledge of previous researches showing that anxiety is closely related to skills to manage stress (Rahnama et al., 2017; Jung et al., 2019; Kaplánová, 2019a, c; Morales-Rodríguez and Pérez-Mármol, 2019).

In the present study, financially awarded football players outperformed football players without financial awards when they scored higher in the subscale coachability. This is the first evidence to show that financial awards can influence the perception of coach criticism and improve his authority. One of the qualitative studies analyzing strategies to maintain a coach–analyst relationship in professional football using the COMPASS model has demonstrated the dominance of coaching attitude in the case of 66.9% football players (Bateman and Jones, 2019). In our study, we also demonstrated that financially awarded football players are humbler than unawarded football players; they are better at accepting constructive criticism from the coach without taking it personally or becoming upset. It seems that professional football players prefer an authoritative coach approach that increases their mental resilience, which is consistent with partial findings (Géczi et al., 2008). Although the results suggest that funding has a positive impact on the development of players’ mental resilience and the coach can be an important facilitator for their improvement, our study also revealed that funding increases the anxiety of football players’ cognitive trait anxiety, which is manifested by increased fear of failure and inhibits sports performance (Kaplánová, 2019b). There are various techniques used by sports psychologists to reduce cognitive trait anxiety. Perhaps the most effective is a cognitive technique called reframing. Reframing helps coaches transform the negative content in the athlete’s mind into a more positive form. It is the framing of negative thoughts that hamper sports performance into a perspective that promotes sports performance. This technique allows athletes to look at the situation from a different perspective, helping to see the wider context; it allows them to recognize and appreciate a positive view even in a seemingly negative situation (Kaplánová and Gregor, 2019). Reframing does not change the athletes’ situation; it only changes their emotional experience by pointing out the importance of not giving up (Mattila, 2015). Therefore, we encourage sports organizations to train football coaches under the supervision of sport psychologists with a special focus on developing the relationship between the coach and football players, as well as using effective cognitive techniques, e.g., reframing, to reduce cognitive trait anxiety.

Mental training is an essential part of the complex training of athletes. The correct setting of short- and long-term goals is very important for athletes (Bebetsos and Antoniou, 2003; Daroglou, 2011; Bebetsos, 2015). Athletes feel regret, sadness, and disappointment after a lost match, and according to Higgins’ self-discrepancy theory, this can be explained by the contradiction between the real and the expected self (Bak, 2014; Kaplánová, 2019a, b,c). If an athlete’s performances in competition are lower than their aspirational level, it is a form of failure for the athletes, saturated with the emotions of hopelessness, regret, and grief. When there is insufficient satisfaction of the need for success or coping with a critical situation, the athlete experiences frustration (Kaplánová and Gregor, 2019). In our research, it seems that financially awarded football players have achieved higher scores in subscales goal setting and mental preparation compared to football players without financial awards. The gap between expectations and the achievement of goals leads to less frustration and better mental preparation. At the same time, there is a conflict that is likely to increase the cognitive trait anxiety of awarded football players. This conflict can be explained by Dweck’s implicit theories (Dweck, 2012; Costa and Faria, 2018). According to these theories, football players have a strong need for growth, self-development, and self-realization, but at the same time, they wish to satisfy the need for safety. On this basis, it can be assumed that the need for self-realization in the context of sport satisfies the excellent sports performance of an individual or a team. However, the need for safety is provided to the audience. Audience’s emotions seem to be an important factor for the performance of professional football players (Ribeiro et al., 2017). Therefore, we think that by implementing psychological training for football players, which will also deal with conflicts of needs, we can increase their sports performance while reducing the frustration of the audience from poor team performance.

The moderation effect of financial awards was detected as significant between the subscale confidence and achievement motivation and cognitive trait anxiety. Confidence is made up of self-assessment of one’s own activities, the outcome of the social comparison process with others, and a reflection of fan, audience, or media support (Keegan et al., 2010; Harwood et al., 2015; Kaplánová and Gregor, 2018; Bojanić et al., 2019). If the environment is favorable and supportive, it is manifested by increasing confidence and the motivation to achieve success of athletes; if the environment is hostile, it reduces the performance (Hays et al., 2009; Kaplánová, 2019b). The results of our study showed that financially awarded football players have higher confidence compared to the other unawarded group and that financial awards positively influence achievement motivation. Signing a contract with a football club creates a psychological commitment that can be defined as an internal psychological state of mind that an individual has against an object (Heere and Dickson, 2008). In the context of sport, this may be a football club that expects athletes to have excellent sports performance but also fans and audiences who came to support the preferred football team. Keeping this commitment and avoiding sanctions can increase football players’ cognitive trait anxiety, manifested by negative thoughts and fear of losing financial benefits, popularity, or prestige, which extends the findings of previous studies (Kaplánová, 2019b, c). There are studies confirming that a high level of confidence improves athletes’ sports performance (Kaplánová and Gregor, 2018; Kaplánová, 2019b), but even those consider that too high a level of confidence may be an obstacle to performance (Hays et al., 2009). Therefore, we recommend that sports organizations also undertake training courses for coaches and football players aimed at developing adequate confidence and achievement motivation, as well as courses aimed at dealing with conflicts caused by pressure from fans, audiences, or media on confidence of football players. The aim of psychological training of athletes should be to ensure adequate psychological adaptation of athletes not only to training but also to competitive conditions in sport. For this reason, in the short term, we recommend that sports psychologists focus their psychological training on regulating the current mental state of football players (precompetitive, competitive, and postcompetitive states) in order to achieve their maximum sports performance. In long-term psychological training, we recommend focusing on increasing the psychological resilience of football players by shaping their personality traits, which is a demonstrable prerequisite for high performance in sports and requires a continuous psychological approach under the guidance of a trained sports psychologist.

The limit of the study is the investigation of the moderating effect of financial awards on the relationship between football players’ anxiety and coping skills only in the male gender. For this reason, we recommend expanding the research sample to include female football players in future research or conducting a gender comparison study. Another limit of the study is the different values of salaries of football players, which may have played a different role in experiencing anxiety. Therefore, we recommend extending research to this aspect as well.

## Conclusion

The results evidenced the fact that financial awards are important factors affecting the cognitive trait anxiety of football players and their selected coping skills. Financial awards increase confidence, achievement motivation, coachability, goal setting, and mental preparation of football players, which proves them to be supportive of sports performance but also increases cognitive trait anxiety, which ties with negative thoughts inhibiting performance in sports. For this reason, we encourage sports organizations to focus more on the mental preparation of football players. We recommend football players graduate from short- or long-term mental training conducted by a trained sports psychologist not only at the time of their failure but also as a preventive measure against increasing cognitive anxiety. We recommend sports organizations to train coaches in the field of mental training to influence the development of desirable athletes’ coping skills.

## Data Availability Statement

The raw data supporting the conclusions of this article will be made available by the authors, without undue reservation, to any qualified researcher.

## Ethics Statement

The studies involving human participants were reviewed and approved by the Comenius University in Bratislava, Slovakia. The patients/participants provided their written informed consent to participate in this study.

## Author Contributions

AK contributed conception and design of the study, organized the database, performed the statistical analysis, wrote the first draft of the manuscript, contributed to manuscript revision and, read and approved the submitted version.

## Conflict of Interest

The author declares that the research was conducted in the absence of any commercial or financial relationships that could be construed as a potential conflict of interest.
